# The aryl hydrocarbon receptor pathway: a linking bridge between the gut microbiome and neurodegenerative diseases

**DOI:** 10.3389/fncel.2024.1433747

**Published:** 2024-08-08

**Authors:** Lorena Coretti, Elisabetta Buommino, Francesca Lembo

**Affiliations:** ^1^Department of Pharmacy, University of Naples Federico II, Naples, Italy; ^2^Task Force on Microbiome Studies, University of Naples Federico II, Naples, Italy

**Keywords:** microbiota-gut-brain axis, neuroinflammation, AHR, Alzheimer’s disease, Parkinson’s disease, tryptophan metabolism

## Abstract

The Aryl hydrocarbon receptor (AHR) is a cytosolic receptor and ligand-activated transcription factor widely expressed across various cell types in the body. Its signaling is vital for host responses at barrier sites, regulating epithelial renewal, barrier integrity, and the activities of several types of immune cells. This makes AHR essential for various cellular responses during aging, especially those governing inflammation and immunity. In this review, we provided an overview of the mechanisms by which the AHR mediates inflammatory response at gut and brain level through signals from intestinal microbes. The age-related reduction of gut microbiota functions is perceived as a trigger of aberrant immune responses linking gut and brain inflammation to neurodegeneration. Thus, we explored gut microbiome impact on the nature and availability of AHR ligands and outcomes for several signaling pathways involved in neurodegenerative diseases and age-associated decline of brain functions, with an insight on Parkinson’s and Alzheimer’s diseases, the most common neurodegenerative diseases in the elderly. Specifically, we focused on microbial tryptophan catabolism responsible for the production of several AHR ligands. Perspectives for the development of microbiota-based interventions targeting AHR activity are presented for a healthy aging.

## 1 Introduction

The Aryl hydrocarbon receptor (AHR) has been identified as a crucial point of convergence among various cellular signaling pathways implicated in aging, as those governing inflammation and immunity in both the gut and the brain, as well as cell proliferation and differentiation. This is especially relevant in the context of inflammaging and neurodegenerative diseases. There is a growing recognition of the gut microbiota’s role in initiating chronic inflammation by disturbing intestinal balance. Numerous cross-sectional studies have highlighted alterations in microbiota composition among patients with neurodegenerative conditions such as Alzheimer’s (AD) and Parkinson’s diseases (PD), compared to healthy individuals. AD is a multifactorial disease where neuroinflammation, accumulation of beta-amyloid (Aβ) plaque, and neurofibrillary tau tangles in the brain are the causative agents of the progressive loss of memory, language, and cognitive ability in affected individuals (Alzheimer’s Association, 2024). In PD, the progressive loss of neurons in the substantia nigra pars compacta results in dopamine depletion in the dorsal aspect of the putamen, a part of the basal ganglia. This depletion gives rise to the most prominent signs and symptoms of PD, including unintended or uncontrollable movements, such as shaking, stiffness, and difficulties with balance and coordination (Simon et al., 2020). The specific mechanisms and molecular pathways linking gut microbiota alteration and neurodegenerative diseases still remain in their infancy in terms of comprehensive understanding. The AHR has been frequently implicated as a target and mediator of metabolites derived from commensal microbes, in particular those resulting from tryptophan (trp) catabolism (Barroso et al., 2021). Recent research studies evidence that the AHR might serve as a crucial link between dysfunctional microbiota and neurodegeneration both through its direct activation in CNS resident cells or by AHR contribution to peripheral modulation of immune cells and inflammatory responses.

The recognition of AHR pathways modulation upon microbial stimulation and its impact on cellular and tissue balance in processes encompassing neurodegenerative phenotype development may orientate the identification of effective and innovative microbiota-based interventions in the treatment of neurodegenerative diseases.

## 2 AHR in the regulation of immune/inflammatory response in gut and brain

The gut-brain axis has emerged as a critical area of study, shedding light on the intricate bidirectional communication between the gastrointestinal system and the central nervous system (CNS). This interaction is considered a key factor in the pathogenesis and progression of several neurological disorders, including neurodegenerative diseases. Within this context, the AHR has garnered significant attention due to its pivotal role in modulating immune responses, barrier function, and neurotransmitter production. CNS-resident cells harbor the AHR; immune and inflammatory pathways, triggered peripherally by AHR activation, can travel to the brain and affect its physiology. Here, we will describe the involvement of AHR in immune/inflammatory response along this axis.

The AHR is a cytosolic receptor and a ligand-activated transcription factor widely expressed by different cell types throughout the body. AHR signaling is considered a key component of the immune response at barrier sites, regulating epithelial renewal, barrier integrity, but also the activities of many immune cell types, as intraepithelial lymphocytes (IELs), T helper 17 cells (Th17), macrophages and others (Lamas et al., 2018). All these functions make AHR crucial for intestinal homeostasis, where it modulates physiological processes in response to environmental toxins (i.e., 2,3,7,8-tetrachlorodibenzo-p-dioxin and polycyclic aromatic hydrocarbon compounds), endogenous ligands (i.e., 6-formylindolo[3,2-b] carbazole and leukotrienes), and many trp metabolites including those processed by gut microorganisms (Safe et al., 2018).

**Figure 1:**
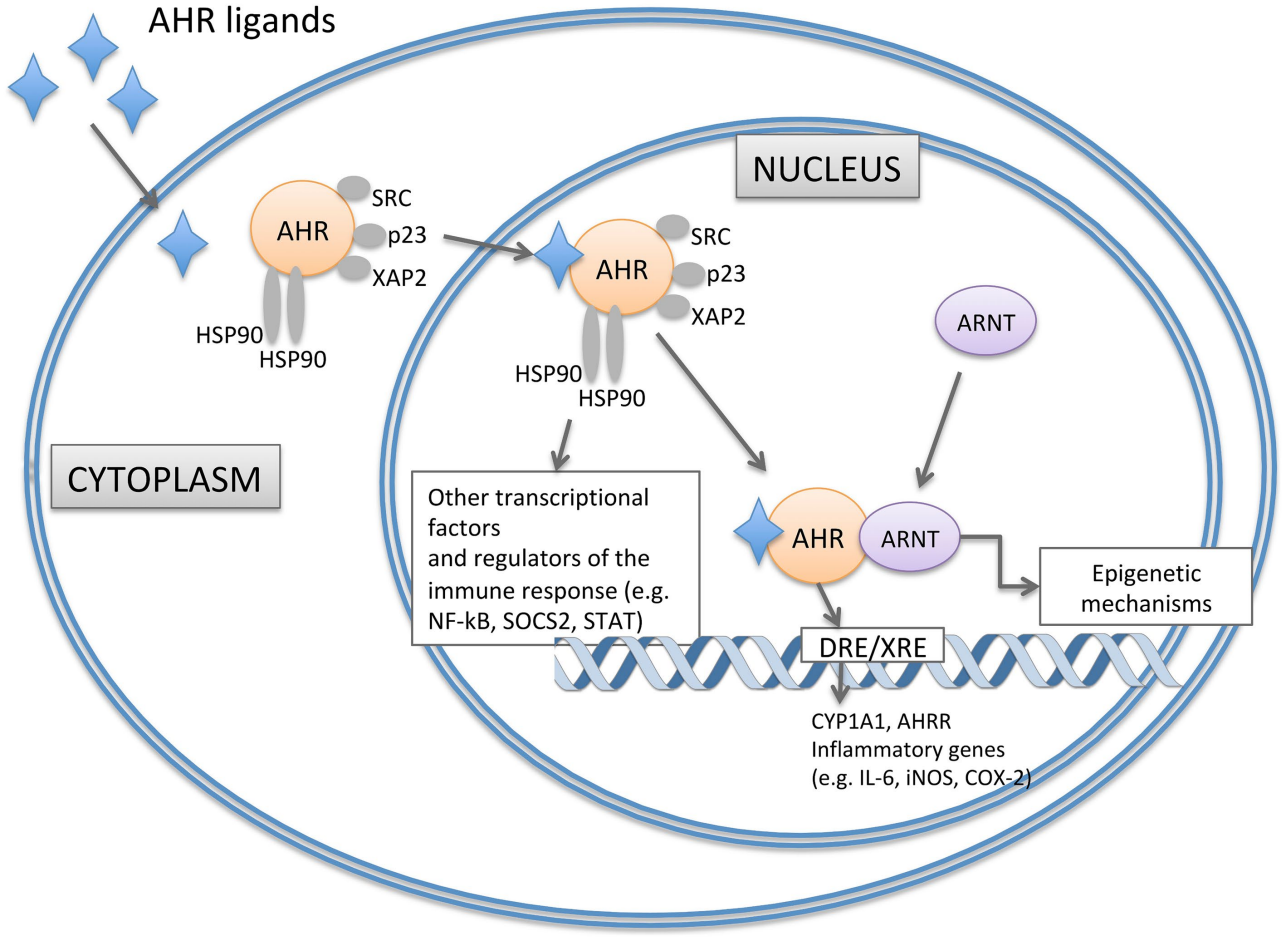
Representation of signaling pathways of AHR. In the absence of ligand, AHR is present in the cytoplasm in an inactive state forming a complex with two HSP90, XAP2, the cochaperone p23 and the protein kinase SRC. Upon ligand binding, AHR translocases to the nucleus and dimerizes with ARNT. The heterodimer interacts with specific DNA regions containing DRE sequences in the regulation site of AHR-responsive genes (such as CYP1A1, AHRR, IL-6, iNOS and COX-2). AHR can also impact the transcription of genes that do not harbour DRE/XRE consensus sequences by epigenetic mechanisms and the interaction with other transcriptional factors (such as NF-kB). AHRR, AHR Repressor; ARNT, AHR Nuclear Translocator; COX-2, Cyclooxygenase-2; CYP1A1, Cytochrome P450 1A1; DRE/XRE, Dioxin/Xenobiotic response element; HSP90, Heat Shock Protein 90; iNOS, Inducible Nitric Oxide Synthase; NF-κB, Nuclear Factor-κB; SOCS2, Suppressor of Cytokine Signaling 2; STAT, Signal Transducer and activator of Transcripton; XAP2, X-Associate Protein 2.

AHR is present in the cytoplasm in a transcriptionally inactive state highly affine to its ligands, forming a complex with two heat shock protein 90 (HSP90), the X-associated protein 2 (XAP2), the cochaperone p23 and the protein kinase SRC. Upon ligand binding, AHR is activated and translocates to the nucleus where dimerizes with the AHR nuclear translocator (ARNT) forming a functional DNA-binding transcription factor (Figure 1). This heterodimer interacts with specific DNA sequences (dioxin/xenobiotic response element - DRE/XRE) in the regulation site of AHR-responsive genes leading, among the other events, to the degradation of AHR ligands (Figure 1). By interacting with other transcriptional factors and co-activators, AHR can also impact the transcription of genes that do not harbor DRE/XRE consensus sequences (recently reviewed by Sondermann et al., 2023). Additionally, AHR is also involved in epigenetic mechanisms, e.g., by controlling histone acetylation and methylation, long non-coding RNA, or microRNAs (Schnekenburger et al., 2007; Chang et al., 2014; Liu et al., 2018; Figure 1).

Various genes associated with inflammatory responses contain multiple DREs in their upstream sequences (Figure 1). Notable examples include the inflammatory mediators IL-6, the inducible nitric oxide synthase and cyclooxygenase-2 (Hollingshead et al., 2008; Lee Y. et al., 2015). Finally, AHR also interacts with the nuclear factorκB (NFκB) that is involved in the expression of pro-inflammatory and cell survival genes (Figure 1). AHR regulates NFκB signaling directly by interacting with RELA, RELB and other members of the NFκB pathway and indirectly through suppressor of cytokine signaling 2 (SOCS2)dependent mechanisms. Similarly, an intricate crosstalk of reciprocal control has been reported between AHR and the signal transducer and activator of transcription (STAT), crucially involved in the immunoregulation (Sondermann et al., 2023). Thus, AHR can switch from the pro-inflammatory to the anti-inflammatory activity playing different roles in the regulation of immune responses that are extremely important at epithelial barriers, such as the intestinal epithelial barrier (Esser and Rannug, 2015). In the context of the gastrointestinal tract, AHR is expressed in immune, epithelial, endothelial, and neuronal cells. Here, its role is to influence different aspects of intestinal barrier function, such as the intestinal epithelial cells (IECs) renewal and turnover, development, function and maintenance of mucosal immune system, and colonic peristalsis. Mice with genetic deletion of AHR show impaired proliferation of colonic crypt stem cells (Han et al., 2020); the specific deficiency of AHR at the intestinal epithelium results in enhanced apoptosis of epithelial cells in a mouse model of Dextran Sodium Sulfate (DSS)-induced intestinal inflammation (Chinen et al., 2015). The influence on intestinal epithelium can also derive from the expression and activation of AHR in immune cells residing in the gut, impacting on their ability to maintain barrier function and protection against infective insults. Different intestinal cells of the innate and adaptive immune response, such as IELs, Th17 cells, innate lymphoid cells (ILCs), macrophages, dendritic cells (DCs), and neutrophils, express AHR (Stockinger et al., 2021). Under homeostatic conditions, AHR activation through specific ligands from the diet or microbiota metabolism is crucial for preserving the integrity and functionality of the intestinal barrier. This signaling results in the production of IL-22, the induction of IL-10 receptor expression, reinforcement of tight junctions, and impacts on colonic neurons. IL-22, predominantly produced by type 3 innate lymphoid cells (ILC3s) under steady-state conditions, induces the secretion of antimicrobial peptides (for example, RegIIIbeta and RegIIIgamma) by IECs for barrier protection (Liang et al., 2006). AHR is also highly expressed in intestinal FoxP3+ Treg cells that balance the tissue inflammation by secreting IL-10 and TGF-beta. On contrary, a lack of AHR leads to an imbalance in immune cell populations, an increase in pro-inflammatory cytokines [tumor necrosis factor (TNF), IL-6, IL-17, and interferon-gamma (IFNγ)], vascular permeability, reduced mucus layer, and disruption of the barrier, including impaired tight junctions’ expression (Ye et al., 2017; Yu et al., 2018). Recently, AHR expression has also been identified in the colonic enteric nervous system (ENS) where the loss of *Ahr* results in delayed transit time that can lead to bacterial overgrowth and chronic constipation (Obata et al., 2020).

AHR is also widely expressed in the CNS from several neuronal cell types, including brain microvessels, neurons, astrocytes and microglia, where it modulates their activity during neurodevelopment and neuroinflammation (Juricek and Coumoul, 2018). Microglia and astrocytes play an important role in the control of the inflammation in the CNS by sensing and coordinating the reactions to endogenous and environmental stimuli, including AHR ligands. Thus, AHR can mediate pro-inflammatory and anti-inflammatory effects in the CNS depending on the availability of exogenous AHR ligands (Lee Y. et al., 2015). Noteworthy, deletion of AHR in both astrocytes and microglia is associated with dysregulated pro-inflammatory response and worsening of inflammatory demyelinating disease (Rothhammer et al., 2018). A central role in CNS inflammation is played by the complex interactions between the AHR and NF-κB that impact on the signaling between astrocytes and microglia. In both glial cells the activation of AHR by its ligands limits NF-κB activation in a SOCS2-dependent manner suppressing NF-κB control of glial responses, as demonstrated in a model of experimental autoimmune encephalomyelitis (EAE). Rothhammer et al. demonstrated that, in the EAE mouse model, deletion of AHR in microglia triggered exaggerated inflammatory responses in local astrocytes, thus microglial AHR expression limits pathogenic activities of astrocytes in chronic autoimmune inflammation (Rothhammer et al., 2018).

In summary, AHR plays a pivotal role in maintaining the integrity and function of the gut barrier, regulating immune responses, and modulating neuroinflammation in the brain. Peripheral homeostasis modulated by AHR can affect CNS resident cells and brain inflammatory processes, warning a critical mediator role for AHR in the gut-brain axis.

Altogether the activation of AHR at peripheral and central level upon different signals is a hotspot of modulation of inflammatory response as well as crucial to govern inflammatory route of the bidirectional communication between gut and brain.

## 3 Age-related microbiota changes and neurodegenerative diseases

Inflammaging is an excessive inflammatory process occurring during aging that significantly influences the development of various age-related diseases. At central level, persistent neuroinflammation can flow into the development of neurodegenerative disorders such as AD and PD (Kempuraj et al., 2015; Bostanciklioğlu, 2018). Several pathophysiological mechanisms have been considered for these neurodegenerative diseases, encompassing genetic, environmental, and endogenous factors that sustain persistent inflammatory responses in the brain, including peripheral inflammation triggered by gut dysbiosis. In fact, a growing body of evidence links the neuroinflammation observed in AD and PD with alteration in gut microbiota composition, suggesting a role for microbial-derived metabolites and host-related responses in the pathogenesis and progression of the diseases.

Changes in the composition of the gut microbiota occur in elderly and are associated with increased inflammation in various tissues and organs including brain tissue (Sarkar and Pitchumoni, 2017; Giau et al., 2018). Hallmarks of aging include genetic instability, cellular senescence, augmented oxidative stress, an imbalance of key messengers (as decline in growth factors), and finally, physiological changes in the gastrointestinal tract. In the intestine, these changes include hypochlorhydria, gastric motility disorders, malabsorption, diarrhea or constipation, degenerative changes in the ENS, all together eliciting dramatic effects on the composition and function of the gut microbiome, whose stability deteriorates in old age (Haran and McCormick, 2021). Many triggering factors as modified diets, use of drugs and reduced physical activity can impact on the gut microbiota of older individuals. A decreased diversity (Biagi et al., 2010), enrichment in pathobionts, and a reduction of bacteria with anti-inflammatory and immunomodulatory properties such as *Bifidobacterium*, *Bacteroides*, *Lactobacillus*, and *Akkermansia* have been reported (Biagi et al., 2016; Ruiz-Ruiz et al., 2019). From a functional perspective, a link between aging and the reduction of microbial pathways associated with trp, and indole production and metabolism has been proposed. Ruiz-Ruiz et al., by shotgun proteomics identified functional microbiome deficits associated with aging. They found that the synthesis of proteins involved in trp and indole production and the fecal concentrations of trp metabolites were progressively decreased with age (Ruiz-Ruiz et al., 2019). The trp and its metabolites are known to play fundamental roles in health and neuroprotection. Their production and catabolism have been found decreased in patients with a number of disorders, including neurodegenerative diseases, such as AD and PD (Favre et al., 2010; Vujkovic-Cvijin et al., 2013; Sandgren and Brummer, 2018; Platten et al., 2019; Chojnacki et al., 2020; Song et al., 2023).

### 3.1 Alteration of gut microbiota profiles in Alzheimer’s disease

The increase of specific bacterial species has been associated to AD dementia, including taxa known to cause inflammatory states, such as the gram negative *Bacteroides vulgatus* (Haran et al., 2019). Haran et al. identified a dysbiotic pattern among AD elders in comparison to those without dementia or with other dementia types. They reported a reduction in species producing short-chain fatty acids (SCFAs), with an increase in species known to have associations with either neurological disorders via inflammation or other colonic inflammatory states (Haran et al., 2019). Lower levels of SCFAs can affect intestinal barrier integrity and permeability allowing the entry of pro-inflammatory bacterial components, like lipopolysaccharide (LPS), into the systemic circulation. LPS, reaching the brain, activates microglia and can promote neuroinflammation and neurodegeneration.

Among the SCFAs, butyrate is the primary microbial metabolite in the gut, serving as a key energy source for colon cells. It binds to cell membrane receptors activating downstream signaling pathways or enters the cells and directly affects gene expression by inhibiting histone deacetylases. In addition to its local effects in the colon, such as maintaining gut barrier integrity and providing anti-inflammatory benefits, butyrate can also influence microbial signaling to the brain through various pathways. Butyrate impacts systemic inflammatory and gastrointestinal endocrine responses, can cross the blood–brain barrier (BBB), and communicates with the brain via vagal afferents (Stilling et al., 2016). By interacting with nearly all systems involved in gut-brain communication, reduced levels of butyrate can likely be linked to AD processes by affecting synaptic plasticity, amyloid-beta and tau pathologies, and neuroinflammation (Qian et al., 2022). Accordingly, the microbiota composition of AD elders is characterized by lower proportions of key butyrate-producing bacteria, such as members of *Butyrivibrio* (*B. hungatei* and *B. proteoclasticus*) and *Eubacterium* (*E. eligens*, *E. hallii*, and *E. rectale*) genera, as well as *Clostridium* sp. *strain SY8519*, *Roseburia hominis*, and *Faecalibacterium prausnitzii*. Moreover, decreased levels of *Bifidobacterium*, *Bacteroides*, *Lachnospira*, and *Ruminiclostridium_9* and increased abundances of *Prevotella* have also been observed in patients with AD (Vogt et al., 2017; Guo et al., 2021). Experimental approaches modulating gut microbiota composition are defining functional roles for gut microbial communities in the progression of AD pathology but are also indicative of a possible therapeutic approach to ameliorate AD symptoms. Depletion of gut microbiota both in germ-free and antibiotic-treated transgenic AD mice induces reduction of cerebral Aβ amyloid pathology and neuroinflammation (Minter et al., 2016; Harach et al., 2017); in the ADLPAPT transgenic mouse model of AD, fecal microbiota transplantation from WT mice ameliorated the AD-like pathology in recipient mice, reversing also peripheral abnormalities related to intestinal macrophage activity and circulating blood inflammation (Kim M. S. et al., 2019). Multiple studies involving different AD rodent models have shown that probiotic interventions remodeled gut microbiota profile, SCFAs levels, inflammatory markers, and cognitive functioning (reviewed in de Rijke et al., 2022). Randomized, double blind, controlled trials with subjects diagnosed with AD found significant improvements in cognition after probiotic administration (Akbari et al., 2016; Tamtaji et al., 2019a). Beneficial effects of prebiotic and symbiotic consumption exerting healthy responses on gut microbiota and its metabolic products have also been recorded (Hoffman et al., 2019; Westfall et al., 2019; Wu et al., 2020; Xu et al., 2020; Deng et al., 2021; Gu et al., 2021; Lee et al., 2021; Liu et al., 2021).

### 3.2 Alteration of gut microbiota profiles in Parkinson’s disease

A pro-inflammatory gut microbiota, rich in gram negative bacteria, source of LPS, and deficient in anti-inflammatory SCFA-producing bacteria has been identified in patients with PD. Although there are conflicting results, the PD-associated microbial profile seems to be characterized by increased relative abundance of Enterobacteriaceae, *Akkermansia*, *Lactobacillus*, *Bifidobacterium*, *Clostridium*, and Ruminococcaceae, along with decreased abundance of Prevotellaceae, Lachnospiraceae and *Faecalibacterium prausnitzii*, the latter being a putative SCFA-producing species (Scheperjans et al., 2014; Keshavarzian et al., 2015; Hill-Burns et al., 2017; Aho et al., 2019; Li et al., 2019). On the other hand, probiotics interventions improve gastrointestinal symptoms of PD patients, such as abdominal pain, abdominal distension and constipation together with markers of inflammation and cognitive impairment (Barichella et al., 2016; Georgescu et al., 2016; Borzabadi et al., 2018; Tamtaji et al., 2019b; Tan et al., 2021). Several animal models of PD have also highlighted the important role of gut microbiota in influencing PD symptoms, progression, and treatment success. Bacteria of genus *Desulfovibrio* induced alpha-synuclein aggregation in the head region of a *Caenorhabditis elegans* model of PD. Noteworthy, some *Desulfovibrio* strains, isolated from PD patients, were more competent to induce alpha-synuclein aggregation compared to strains collected from healthy individual (Huynh et al., 2023). Recently, we have demonstrated that antibiotic-induced gut dysbiosis is able to worsen disease symptoms in a mouse model of PD. Reduction of bacterial diversity, of butyrate-producing bacteria and of fecal butyrate levels along with *Ruminococcus lactaris* increase were the main features of the gut microbiota of these mice. Notably, treatment with butyrate restored gut dysbiosis, gut permeability, positively impacting on motor symptoms and peripheral and central inflammation (Avagliano et al., 2022; Turco et al., 2023).

Collectively these findings, even though mostly based on preclinical and cross-sectional studies, indicate that reconfiguration of gut communities during aging instigate toward elevate peripheral and central inflammation that are associated to the development of neurodegeneration in AD and PD. This picture prompts future investigations on both specific mechanisms and molecular pathways underlying these associations and the consideration of microbiota-targeted approaches for the improvement of clinical outcomes.

## 4 Gut microbiota-derived tryptophan metabolites as ligands for AHR activation: relevance for gut-brain signaling

In the human body, the gut can accommodate an abundance of AHR ligands some of which are directly processed by commensal microorganisms. As such, there is much interest in the involvement of AHR as an integrator of microbial signals at intestinal and central levels to unveil gut-brain axis perturbation in neurodegenerative diseases. As mentioned above, AHR signaling participates in the gut-brain axis through multiple mechanisms ranging from the direct activation of AHR in CNS resident cells to AHR mediated peripheral modulation of inflammation. In the context of aging and neurodegenerative diseases, AHR ligands produced by gut microorganisms’ trp catabolism are under specific attention.

In the next paragraph, we will analyze the impact of microbiota-derived trp metabolites on gut-brain axis through AHR signaling. Specifically, we will focus our attention on some trp metabolites with specific neuroactive features, whose imbalance or deficiency could be involved in the pathophysiology of aging-related brain diseases in the context of AHR signaling pathways and regulatory functions (Table 1).

**Table 1:** The role of tryptophan metabolites in gut-brain axis via AHR activation.

Pathway	Metabolite	Source	AHR Activation in Gut	AHR Activation in Brain	References
Indole	Indole	Gut microbiota (e.g., *Bacteroides* spp., *Clostridium* spp., *E. coli*, *Desulfovibrio vulgaris*)	Modulates local inflammation; maintains mucosal homeostasis	Neuroprotective effects; activates AHR in CNS-resident cells (astrocytes, microglia)	Rothhammer et al. (2016, 2018), Roager and Licht (2018)
	Tryptamine	*Clostridium sporogenes*, *Ruminococcus gnavus*	Immunomodulation	Neuroprotective effects: impacts Treg/Th17 balance	Roager and Licht (2018), Dopkins et al. (2021)
	Indole-3-propionic acid (IPA)	*Clostridium* spp., *Peptostreptococcus* spp.	Maintains intestinal barrier; anti-inflammatory properties	Neuroprotective effects	Rothhammer et al. (2016, 2018), Roager and Licht (2018), Sun et al. (2022)
	Indole-3-acetic acid (IAA)	Gut microbiota (e.g. *Bacteroides* spp., *Bifidobacterium* spp., *Clostridium* spp.)	Maintains intestinal barrier; anti-inflammatory properties	Neuroprotective effects	Roager and Licht (2018), Sun et al. (2022)
	Indole-3-aldehyde (IAld)	*Lactobacillus* spp.	Promotes barrier function and immune tolerance	Neuroprotective effects	Rothhammer et al. (2016, 2018), Cervantes-Barragan et al. (2017), Roager and Licht (2018)
	Indole-3-ethanol	Gut microbiota	Ameliorates inflammation in colitis models	-	Scott et al. (2020)
	Indole-3-pyruvate	Gut microbiota	Enhances epithelial integrity and antimicrobial defense	-	Zelante et al. (2013), Scott et al. (2020)
	Indolelactic acid (ILA)	Gut microbiota (e.g. *Anaerostipes* spp., *Bacteroides* spp., *Bifidobacterium* spp., *Lactobacillus* spp.)	Regulates Treg/Th17 balance; anti-inflammatory effects	-	Cervantes-Barragan et al. (2017), Roager and Licht (2018)
	Indoleacrylic acid (IA)	*Clostridium sporogenes*, *Peptostreptococcus* spp.	Promotes barrier function; maintains intestinal homeostasis and immune tolerance.	-	Roager and Licht (2018)
	3-methylindole (skatole)	Gut microbiota (e.g. *Clostridium* spp., *Lactobacillus* spp., *Eubacterium* spp., *Bacteroides thetaiotaomicron*, *Butyrivibrio fibrisolvens*)	Regulation of intestinal epithelial function	-	Roager and Licht (2018), Kurata et al. (2023)
	Indoxyl-3-sulfate (I3S)	Liver (from bacterial indole)	Supports AHR signaling in systemic circulation	Neuroprotective effects	Banoglu and King (2002)
Kynurenine	Kynurenic acid (KA)	Host, gut microbiota	Promotes intestinal homeostasis	CNS neuroprotective properties	Giil et al. (2017), Miyamoto et al. (2023)
	Quinolinic acid (QA)	Host, gut microbiota	-	Neurotoxic effects; contributes to neurodegenerative processes, increases during inflammation	O’Farrell and Harkin (2017), Dehhaghi et al. (2019)
	Xanthurenic acid (XA)	Host, gut microbiota	Promotes intestinal homeostasis	Not specifically noted for CNS effects	Han et al. (2001), Chen et al. (2023)
	Cinnabarinic acid	Host, gut microbiota	Induces AHR-dependent genes that promote intestinal homeostasis	Not specifically noted for CNS effects	Chen et al. (2023)
	3-Hydroxykynurenine (3-HK)	Host, gut microbiota	Modulates immune responses; potential AHR activation in gut	Neuroprotective effects; influences CNS excitotoxicity	Han et al. (2001), O’Farrell and Harkin (2017), Dehhaghi et al. (2019)
	3-Hydroxyanthranilic acid (3-HAA)	Host, gut microbiota	Anti-inflammatory effects; modulates immune response	Neuroprotective effects; modulates neuroinflammation	O’Farrell and Harkin (2017)
Serotonin	5-Hydroxytryptamine (Serotonin, 5-HT)	Gut enterochromaffin cells, brain	Regulates AHR ligand availability; promotes sustained AHR signaling	Influences neurobiology and behavior via gut-brain axis	Yano et al. (2015), Agus et al. (2018), Manzella et al. (2020)

Dietary trp can be transformed into different metabolites following three major pathways in the gastrointestinal tract: the biotransformation of trp in indoles by gut microbiota, the kynurenine pathway, and the serotonin production pathway (reviewed in Agus et al., 2018).

### 4.1 Indole pathway

Through the indole pathway trp is transformed into several molecules, such as indole and its derivatives, many of which are ligands of the AHR able to induce AHR-dependent signaling for modulation of local inflammation or for neuromodulation (Table 1). Indole-producing species are gram negative and positive bacteria (e.g., members of *Bacteroides* and *Clostridium* genera, *E. coli* and *Desulfovibrio vulgaris*) that express the enzyme tryptophanase; as widely reviewed by Roager and Licht (2018), trp is also transformed by gut commensal in the AHR agonists tryptamine (by *Clostridium sporogenes* and *Ruminococcus gnavus*), indolelactic acid (ILA; by *Anaerostipes* spp., *Bacteroides* spp., *Bifidobacterium* spp., *Lactobacillus* spp. Among the others), indoleacrylic acid (IA; by *Clostriudium sporogenes* and *Peptostreptococcus* spp.), indole-3-propionic acid (IPA; by members of *Clostriudium* and *Peptostreptococcus* spp.), indole-3-aldehyde (IAld; by *Lactobacillus* spp.), indoleacetic acid (IAA; by *Bacteroides* spp., *Bifidobacterium* spp. and *Clostridium* spp. among the others), and the 3-methylindole (or skatole; by members of genera *Clostriudium*, *Lactobacillus*, *Eubacterium*, and *Bacteroides thetaiotaomicron* and *Butyrivibrio fibrisolvens* species). Commensal bacterialderived metabolites can be further metabolized by the host into other AHR agonists, such as the indoxyl3sulfate (I3S) formed in the liver starting from the bacterial indole (Banoglu and King, 2002). Evidence showed that indole-producers commensal bacteria might maintain the mucosal homeostasis and affect the immune system in the gut, as well as in systemic circulation and distal organs, through the AHR activation (Table 1). Indole derivatives produced by *Lactobacillus* spp., as ligands of AHR, reprogram naive CD4+ T helper cells into Treg cells (Cervantes-Barragan et al., 2017) and the polarization of Th17 cells (Wilck et al., 2017), keeping the Treg/Th17 balance whose alteration plays an important role in intestinal inflammatory diseases (Kim W. H. et al., 2019; Yan et al., 2020). The immunomodulatory benefits of indole and its derivatives are partly based on the AHR-driven mechanisms in intestinal DCs, IELs, and ILCs (Li et al., 2021) and AHR modulation of the T cell immunity through the alteration of Treg/Th17 cells with Treg dominance (Quintana et al., 2008). Thus, as AHR ligands, indole and some derivatives play a critical role in regulating epithelial integrity and the immune response, including intestinal stem cells (ISCs) and epithelial regeneration, intestinal barrier protection, and antimicrobial defense (Zelante et al., 2013; Cervantes-Barragan et al., 2017; Hendrikx et al., 2018; Hou et al., 2018; Busbee et al., 2020). Indole-3-ethanol, indole-3-pyruvate, and IAld ameliorate morbidity and inflammation of DSS-induced colitis in mice by maintaining the integrity of the apical junctional complex, a major regulator of intestinal permeability (Scott et al., 2020). Moreover, IA was reported to promote barrier function and immune tolerance in DSS-induced colitis mice by inducing the mRNA expression of the AHR target gene Cyp1a1 in the intestinal epithelium and immune cells (Wlodarska et al., 2017). By binding and activating AHR, indoles also promote the expression of IL-10 supporting barrier function (Powell et al., 2020). Maintenance of intestinal homeostasis and an anti-inflammatory state by gut microbes, through indole-dependent AHR activation, beneficially modulates bottom-up players of the gut-brain axis thus affecting brain functionality and behaviour. Gut indoles can also reach the brain, where, interacting with the AHR expressing cells, exert a potent neuroprotective effect (Table 1). For example, indole, I3S, IPA, IAld and tryptamine generated from dietary trp by the different pathways harbored in commensal bacteria can activate AHR signaling in CNS-resident cells, namely astrocytes and microglia, limiting neuroinflammation (Rothhammer et al., 2016, 2018; Dopkins et al., 2021). Recently, Wei et al. showed that microbiota-derived indole elicits also neurogenic effects in the adult mouse hippocampus by AHR activation, being neurogenesis not induced in AHR KO mice (Wei et al., 2021).

### 4.2 Kinurenine pathway

Dietary trp is mainly metabolized toward the kynurenine pathway (KP) by the host enzymes trp 2,3-dioxygenase (TDO) and indoleamine 2,3-dioxygenase (IDO), expressed in liver and other cells (such as intestinal cells, microglia, astrocytes, macrophages, and neuronal cells). The KP is induced in times of stress and/or immune activation, and it is associated with inflammatory response, cancer, and neurological disorders (AD, amyotrophic lateral sclerosis, Huntington disease, and PD), through the production of different neuroactive metabolites (Guillemin and Brew, 2002; Dehhaghi et al., 2019). This catabolism is mediated in the gut by the rate-limiting enzyme IDO1 and leads to the production of kynurenine, and the downstream products such as quinolonic acid (QA), niacin, nicotinamide adenine dinucleotide, and kynurenic acid (KA). Gut microbes encode enzymes homologous to those of the eukaryotic KP and can also produce kynurenine and downstream metabolites with neurotoxic effects, as the 3-hydroxyanthranilic acid (Vujkovic-Cvijin et al., 2013).

The KP is differently activated in the periphery and CNS. Microbial stimuli (as LPS), cytokines, amyloid peptides, and inflammatory molecules (IFNγ) can induce IDO1 in the gut to control host immunity. Elevated concentrations of kynurenines, as in chronic inflammatory diseases, regulate immune homeostasis by acting as AHR ligands and allow the generation of Treg cells, which protect from chronic hyperinflammatory responses (Romani et al., 2008). This can be protective against pathogens insult, where the activation of Treg cells via AHR prevented the infection and significantly reduced clinical signs of *Streptococcus* arthritis (Bessede et al., 2014). Moreover, at intestinal level, KA, xanthurenic acid, and cinnabarinic acid can bind the AHR and induce the expression of AHR-dependent genes that promote intestinal homeostasis (Chen et al., 2023; Table 1). Thus, a decreased production of KA can result in gut barrier imbalance and loss of integrity, with augmented local and systemic inflammation.

Within the CNS the KP is differentially compartmentalized within astrocytes and microglia. The trp uptake and metabolism in astrocytes lead to the production of the KA, reported to have neuroprotective properties in the CNS, due to its action as an antagonist at the N-methyl-D-aspartate receptor (NMDAR; Giil et al., 2017). In microglia, metabolism of trp gives rise to metabolites with reactive oxidative properties including 3-hydroxykynurenine, 3-hydroxyanthranilic acid, and QA that act as agonists at the NMDAR and may contribute to excitotoxicity and neurotoxicity (Table 1). The concentrations of QA in the human brain can increase drastically in inflamed tissues (O’Farrell and Harkin, 2017). QA causes neuronal degeneration, destabilizes the cytoskeleton, triggers pro-inflammatory responses and apoptosis of neurons and astrocytes, as well as disrupts the BBB (O’Farrell and Harkin, 2017). All these neurotoxic effects can be implicated in the pathology of neurodegenerative and neuropsychiatric disorders (Dehhaghi et al., 2019). The gut microbiota may indirectly influence the levels of circulating trp and kynurenine metabolism and consequently those KP metabolites that are AHR agonists. Germ-free and antibiotic treated animals, as result of lack of IDO stimulation upon bacterial TLRs activation, present higher serum trp and lower kynurenine levels along with a tendency for anxiety-like behavior and cognitive deficits (Campbell et al., 2014; Desbonnet et al., 2015). Moreover, as mentioned above, gut microbiota can synthesize kynurenine metabolites: some bacteria harbor an enzyme aspartic transaminase which transaminates kynurenine and 3-hydroxykynurenine to KA (Han et al., 2001); recently, Miyamoto and co-authors unraveled a bacterial trp metabolism gene (EC:1.13.11.11) that collaborates with intestinal cells in the biosynthesis of KA. This metabolite resulted a key element in the recruitment of pathogenic macrophages and the subsequent induction of Th17 cells promoting EAE in the CNS (Miyamoto et al., 2023). Other bacterial products influencing KP are several group B vitamins that are cofactors of some enzymes of KP, and the post-biotic butyrate that has been shown to reduce IDO transcription, thus blocking the kynurenine production (Więdłocha et al., 2021). Finally, lower kynurenine concentrations can also result from decreased trp availability associated with bacterial indole metabolism and serotonin synthesis.

### 4.3 Serotonine pathway

The serotonin (5-hydroxytryptamine, 5-HT) production pathway is the third route of trp metabolism that partially occurs in the brain, while for the 90% in the intestinal enterochromaffin cells via Trp hydroxylase 1 (TpH1) under the control of gut microbiota (Agus et al., 2018.). Gut-derived 5-HT is a key regulator of AHR ligand availability and receptor activation (Table 1). 5-HT is a CYP1A1 substrate that competes with AHR ligands for CYP1A1 degradation. Thus, via the inhibition of the enzymatic clearance of AHR ligands, 5-HT promotes sustained AHR signaling. In accordance, lack in the gut of 5-HT intracellular transport via the plasma membrane 5-HT transporter (SERT) impairs intestinal AHR activation (Manzella et al., 2020). Gut and brain 5-HT levels are correlated to specific members of the gut microbiota (Yano et al., 2015; Pirozzi et al., 2023), thus gut bacteria participate in several physiological functions that depend on 5-HT levels, such as intestinal motility and absorption of nutrients, but also in the underlying neurobiology of different behaviors. Gut microbial communities bidirectionally interact with host 5-HT (reviewed by Everett et al., 2022). Luminal 5-HT levels can favor certain species, some of those can increase 5-HT concentrations by changing the expression levels of genes involved in its synthesis, metabolism, secretion, or transport. Additionally, via the gut-brain axis, microbiota can affect brain 5-HT levels by modulating trp brain influx based on trp bacterial metabolism in the gut, but also influencing expression of 5-HT-related genes at central level. For example, bacterial extracellular vesicles derived from *Akkermansia muciniphila* cause an increase of colonic and hippocampal 5-HT levels, by up-regulating 5-HT synthesis enzymes both in the gut and, probably by crossing the BBB, in the brain. On these bases, changes in 5-HT induced by gut microbiota may contribute to various neurological conditions (Yaghoubfar et al., 2020).

**Figure 2:**
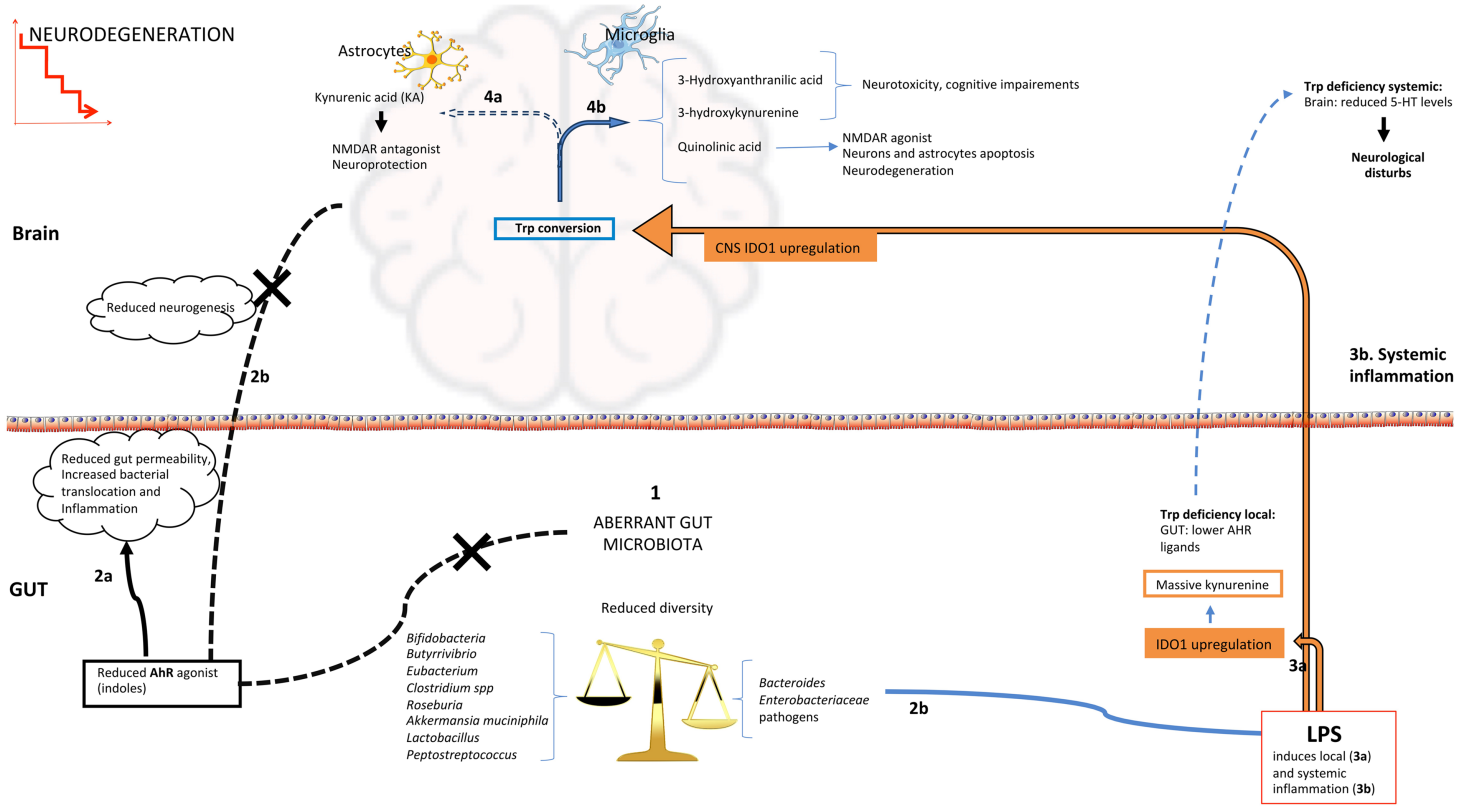
Possible routes of AHR activation in the gut and brain during neurodegeneration. (1) Aberrant microbiota profiles with diversity decline, enrichment in pathobionts and reduction of beneficial bacteria have been associated to neurodegenerative diseases; reduction in indole-producing bacteria induces low indoles levels that are AHR ligands, this can affect both intestinal function (2a; imbalance in immune cell populations, increase in proinflammatory cytokines and permeability), and the immunological state and neurogenesis at central level (2b); increased gut permeability could favour the translocation of inflammatory bacterial components like LPS into the systemic circulation, that reaching the brain and activating microglia can promote neuroinflammation (3b); (2d) gram-negative bacteria are source of LPS that can increase local inflammation and upregulate IDO1 activity increasing the kynurenine pathway of tryptophan degradation (3a) leading to indoles and 5-HT reduction; systemic LPS (3b) reaching the brain affects tryptophan conversion reducing the production of KA in astrocytes (4a) and increasing other downstream metabolites of kynurenine pathway with negative effects on neuronal homeostasis (4b). 5-HT, 5-hydroxytryptamine; IDO1, indoleamine 2,3-dioxygenase 1; KA, kynurenic acid.

Overall, a balance among the three pathways for trp catabolism is essential to maintain intestinal and central homeostasis and functionality. An excess or change in the ratio of trp pathways’ by-products could be responsible for several consequences, including neurological disturbances (Figure 2). For example, IDO1 pathway over-activation by intestinal mononuclear cells, which induces massive production of kynurenine, causes a deficiency of trp in brain and in 5-HT production, with consequent neurological disturbs. The over-activation of IDO1 may also decrease the gastrointestinal availability of trp, contributing to lower production of bacterial AHR agonists. In support of this evidence, the supplementation of trp or trp-derived agonists can limit CNS inflammation (Rothhammer et al., 2016). Based on the bacterial contribution to trp availability for each pathway, this balance strongly depends on gut microbiota membership. Neurodegeneration can be linked to alteration of microbiota structure and composition through modulation of several pathways involving gut microorganisms’ metabolic functions of trp catabolism (Figure 2).

## 5 Convergence of AHR signaling and aging-related brain diseases encompasses trp metabolism derivatives of gut microbiota

Despite a plethora of factors have been recognized as playing an important role in the onset of neurodegenerative diseases, AHR signaling is gaining attention due to the link with gut commensal trp derivatives and the control of CNS inflammation.

We will here consider the convergence of AHR signaling in PD and AD in which chronic inflammation represents a crucial cause of brain cell damage and death. Aberrant microbiota profiles described in patients with AD and PD could promote neuroinflammation by affecting local trp metabolism and AHR-dependent mechanisms that are critical regulators of inflammatory players at both gut and central level.

Abnormal levels of AHR have been found in post-mortem brains and circulation of AD patients (Rothhammer and Quintana, 2019; Ramos-García et al., 2020). Among the key features marking the gut microbiota alteration identified in patients with AD, reduction in species able to produce AHR ligands has also been described. Specifically, *Bifidobacterium*, *Bacteroides*, and several members of Firmicutes phylum, such as *Lactobacillus* spp. that are reduced in patients with AD, are indole-producing bacteria. Notably, long-term dietary supplementation of probiotics containing *Lactobacillus* and *Bifidobacterium* strains showed positive impact on cognitive function, learning and memory in a rat model of AD (Rezaeiasl et al., 2019). Moreover, fecal metabolomics profiling identified a disturbance of trp metabolism in AD patients, particularly related to metabolites processed by gut commensals, being the levels of trp host derivatives kynurenine and KA not changed. Interestingly, metabolites in the serotonin pathway, co-produced by host and gut microbiota, and some microbial indole derivatives, that are AHR ligands, were significantly decreased in AD (Wu et al., 2021). Decreased plasma levels of trp and ILA have also been associated with the progression of AD, suggesting enhanced trp degradation through the kynurenine pathway in AD (Shao et al., 2020). Recently, Sun et al. found abnormal levels of indole-producing bacteria in the APP/PS1 mouse model of AD; they demonstrated that treatment with indole and its derivatives (IAA and IPA) improved gut integrity and, by increasing the levels of AHR, reduced the inflammatory response and cognitive impairment of APP/PS1 mice (Sun et al., 2022). In another study, Pan and co-authors also demonstrated that high-trp diet ameliorates cognitive dysfunction and decreases Aβ deposition in APP/PS1 mice (Pan et al., 2023). Collectively, it is suggested that trp and its indoles derivatives could exert anti-neuroinflammatory effect by activating the AhR and restraining the NF-κB pathway in AD. Regarding KP by-products, whose levels can affect trp, 5-HT and indoles availability (Chatterjee et al., 2019), several evidences reported their abnormal levels both in patients and animal models of PD. Aberrant levels of QA, 3-hydroxykynurenine, and KA together with increased expression of IDO1 have been associated with AD hallmarks (Chatterjee et al., 2019). This increase in the KP intermediates is mainly observed in microglia and astrocytes, which are the mediators of neuroinflammation in AD. The increased secretion of inflammatory signaling molecules can both further trigger Aβ generation and immune responses, and activate IDO1 resulting in increased trp degradation and increased KP metabolites. Thus, the establishment of a vicious circle that encompasses a reduction of gut bacterial indole-producers can underlie the progression of neurodegeneration in AD.

Altered metabolism of trp and AHR activation has also been found in patients with PD and in animal models of the disease. The association between KP metabolites and PD has been extensively reviewed (Venkatesan et al., 2020). In addition, levels of neuroprotective trp metabolites, such as IAA and KA, have been found reduced in patients with PD (Chen and Lin, 2022). Interestingly, dietary trp significantly ameliorates impaired motor function, up-regulates tyrosine hydroxylase expression (the enzyme responsible for dopamine and L-DOPA production), inhibits NF-κB in substantia nigra, and down-regulates the serum levels of pro-inflammatory cytokines in rotenone-induced rat model of PD; these effects are reversed by antibiotic treatment with ampicillin and by the inhibition of AHR pathway (Wang et al., 2021), suggesting a concerted activity between microbial metabolism of trp by gut microbiota and host AHR activation that can protect from rotenone-induced neurotoxicity.

Thus, seminal evidence underlies that impaired production of AHR ligands by the gut microbiota represents an important factor in AD and PD. Given the influence of gut microbiota and its metabolites in preventing local intestinal as well as central inflammation, further investigation should explore how microbiota-based interventions could stimulate AHR to provide its critical functions and their impact on neurodegeneration.

## 6 Conclusions and future perspectives

The literature review emphasizes the significant role of the interaction between gut microbiota, trp metabolism and AHR in regulating both host health and disease. In elderly individuals, alterations in gut microbiota composition, including a decrease in indole-producing bacteria, may contribute to age-related reduction in systemic levels of indoles, affecting AHR signaling. All this can impact the host physiology leading to inflammaging, neuroinflammation, and compromised neuroprotective mechanisms. Thus, future microbiota-based interventions targeting AHR and its ligands show promise in reducing intestinal and CNS inflammation. Sustaining the production of indoles by the gut microbiota through dietary modifications (as dietary trp), prebiotics or probiotic therapies could benefit aging individuals by promoting adult neurogenesis. Additionally, administering peculiar postbiotics that are AHR ligands, namely indoles, could compensate for a suboptimal microbiota lacking indole-producing bacteria. This can represent a personalized therapy to counteract cognitive decline. Beside to be an important mediator in the interkingdom communication between gut bacteria and host by AHR signaling, indole is also an intra- and interspecies signaling molecule that, acting as signal of the quorum sensing, is able to influence the behavior of the gut microbe’s community members (Lee J. H. et al., 2015). This could favor the prevalence of bacterial species indole-responsive able themselves to produce AHR ligands helpful for immune modulation. Thus, indole may play a crucial role in reshaping disrupted gut microbial communities observed in patients with neurodegenerative diseases.

## Author contributions

LC: Writing – original draft. EB: Writing – original draft, Writing – review & editing. FL: Writing – review & editing, Conceptualization.
